# Supplementary material for: Butyric Acid from Probiotic Staphylococcus epidermidis in the Skin Microbiome Down-Regulates the Ultraviolet-Induced Pro-Inflammatory IL-6 Cytokine via Short-Chain Fatty Acid Receptor
Source: Int J Mol Sci. 2019 Sep 11;20(18):4477. doi: 10.3390/ijms20184477 (PMC6769796; doi:10.3390/ijms20184477)
Supplement: Supplementary file 1 [file ijms-20-04477-s001.pdf]

International Journal of Molecular Sciences
MDPI

Article

Butyric acid from probiotic *Staphylococcus epidermidis* in the skin microbiome down-regulates the ultraviolet-induced pro-inflammatory IL-6 cytokine via short-chain fatty acid receptor

Sunita Keshari[1], Arun Balasubramaniam[2], Binderiya Myagmardoloonjin[2], Deron Raymond Herr[3], Indira Putri Negari[2] and Chun-Ming Huang[2,4,*]

[1] Department of Life Sciences, National Central University, Taoyuan 32001, Taiwan; sunitakeshari827@gmail.com
[2] Department of Biomedical Sciences and Engineering, National Central University, Taoyuan 32001, Taiwan; arunb2895@gmail.com; binderya.smile@yahoo.com indiraputri24@g.ncu.edu.tw
[3] Department of Pharmacology, National University of Singapore, Singapore 117600, Singapore; phcdrh@nus.edu.sg
[4] Department of Dermatology, School of Medicine, University of California, San Diego, CA 92093, USA
* Correspondence: chunming@ncu.edu.tw Tel: +886-3-422-7151 (ext. 36101); Fax: +886-3-425-3427

1. Supplementary Information

1.1. Western Blotting

Mouse dorsal skin was collected after 4 weeks of UVB exposure and were lysed with T-PER™ Tissue Protein Extraction Reagent (ThermoFisher Scientific, Waltham, MA, USA) supplemented with an EDTA-Free protease inhibitor cocktail (Sigma-Aldrich). Tissue lysates (20 μg) were subjected to 10% sodium dodecyl sulfate polyacrylamide gel electrophoresis gel (SDS-PAGE), then transferred to a polyvinylidene difluoride (PVDF) membrane (Millipore, Temecula, CA, USA) and blocked with 5% (w/v) non-fat milk before incubation overnight with primary antibodies to FFAR2 (1:1,000, Protein Tech,) and β-actin (1:1,000, Cusabio). This was followed by treatment with horseradish peroxidase (HRP)-conjugated secondary antibody (goat anti-rabbit or anti-mouse (1:5,000), ThermoFisher Scientific) for 1 h. Protein bands were detected with a chemiluminescent detection reagent (ThermoFisher Scientific) and Omega Lum™ C Imaging System (Gel company, San Francisco, CA, USA). Densitometry analysis of protein bands were conducted using ImageJ software.

2.2 Real Time PCR

RNA (100 ng) was converted into cDNA using an iScript cDNA Synthesis Kit (Bio-Rad, Hercules, CA, USA). All sets were designed using National Center for Biotechnology Information (NCBI) Primer-Blast (https://www.ncbi.nlm.nih.gov/tools/primerblast/). The reaction was performed on StepOnePlus Real-Time PCR System (Thermo Fisher Scientific) using Power SYBRGreen PCR Master Mix (Thermo Fisher Scientific). The reaction conditions for 40 cycles are as follows: 95°C for 10 min followed by 95°C for 15 s, 55°C for 60 s, and 72°C for 30 s. All reactions were carried out with 3 biological replicates, and each analysis consists of 3 technical replicates. The expression of GAPDH gene was used for normalization. The levels of relative expression levels were calculated using the cycle threshold ($2^{-\Delta\Delta Ct}$) method. Primers used for FFAR2 and GAPDH were 5′- ACCCAAGAGCAGCTGGATGT-3′ (forward); 5′-AGCGCCAATAACAGAAGATGGT-3′ (reverse) and 5′-TGTGTCCGTCGTGGATCTGA-3′ (forward); 5′-GATGCCTGCTTCACCACCTT-3′ (reverse) respectively.

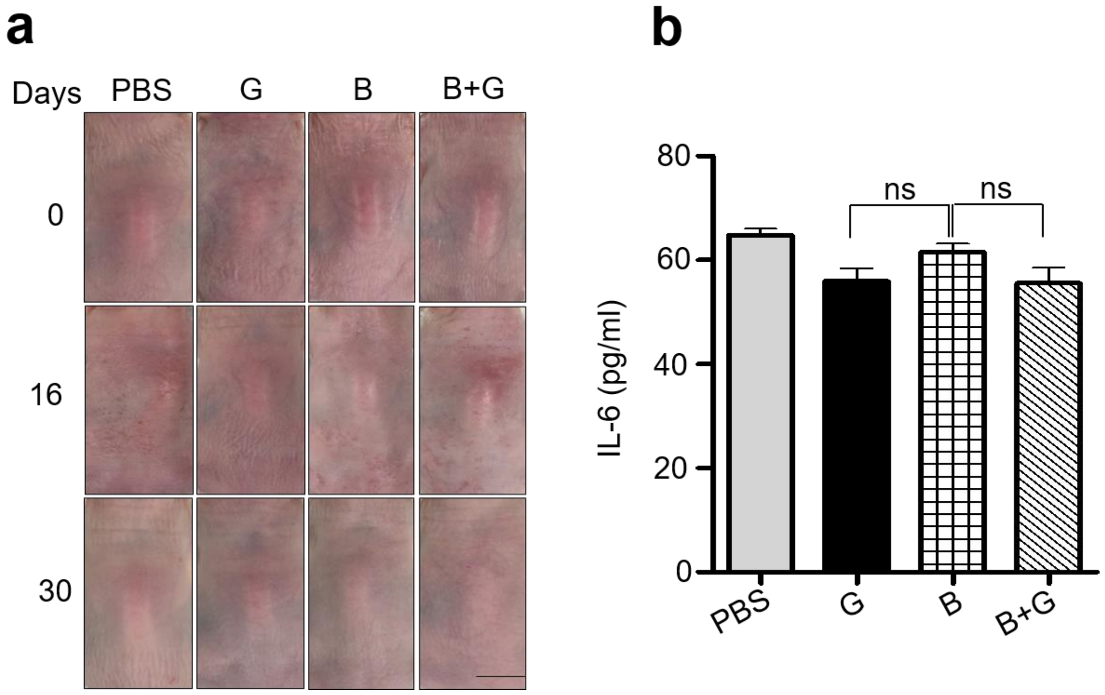

Figure S1. Skin morphology and IL-6 level in mice skin upon application of a mixture of *S. epidermidis* and glycerol. (a) Skin morphology of ICR mice, topically applied with PBS, glycerol (G) (2%), *S. epidermidis* bacteria (B) (10^7 CFU/ml), or mixture of *S. epidermidis* bacteria plus glycerol (B+G) without UVB-irradiation as a control for UVB irradiated groups. Scale bar = 5 mm. Graph (b) indicates the level of IL-6 in skin (c) from above all groups of ICR mice was quantified using a mouse IL-6 enzyme-linked immunosorbent assay (ELISA) kit (R&D Systems). Data are the means of three individual experiments using five mice per group. ns = non-significant. *** $p < 0.001$. (two-tailed t-tests).

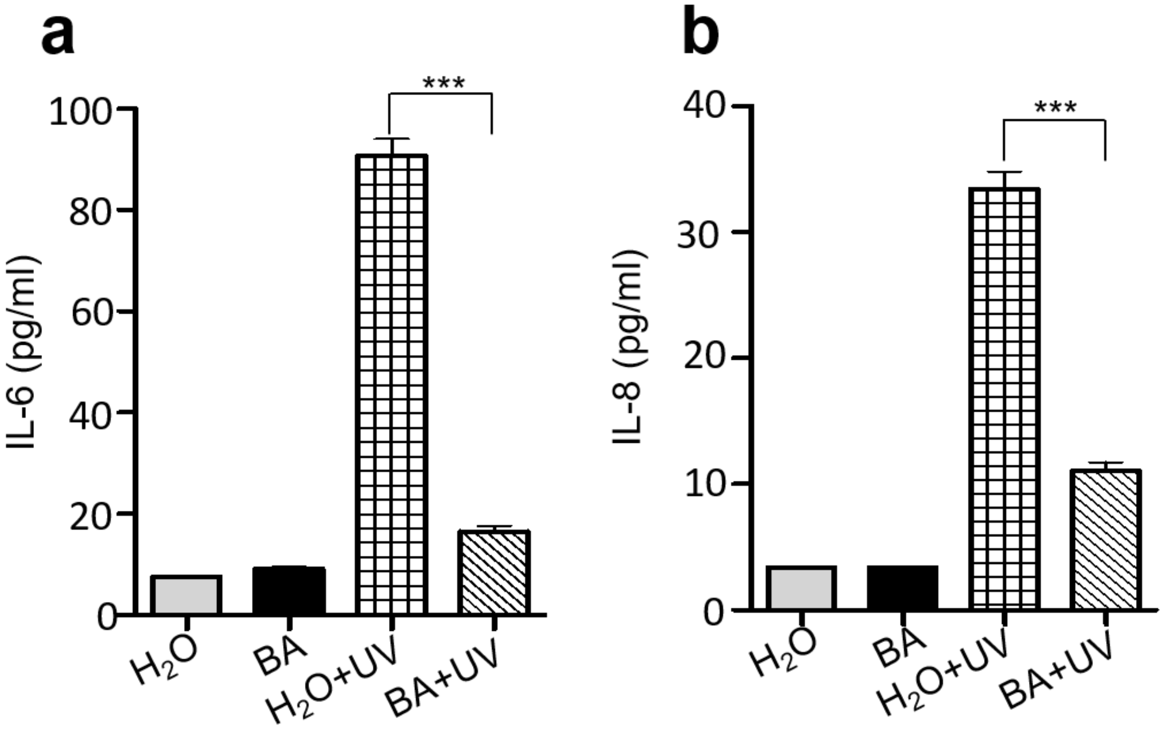

Figure S2. The effect of BA (butyric acid) on IL-6 and IL-8 production in UVB irradiated and non-irradiated human keratinocytes (CCD 1106 KERTr). Human keratinocytes irradiated with UVB (195 mJ/cm2) or non-irradiated were incubated in the presence or absence of BA (butyric acid) (4 mM) for 12 h. The level of IL-6 (a) and IL-8 (b) from the supernatants of human keratinocytes were detected using an ELISA. Data are the means of three separate experiments using five mice per group. * $p < 0.01$. (two-tailed t-tests).

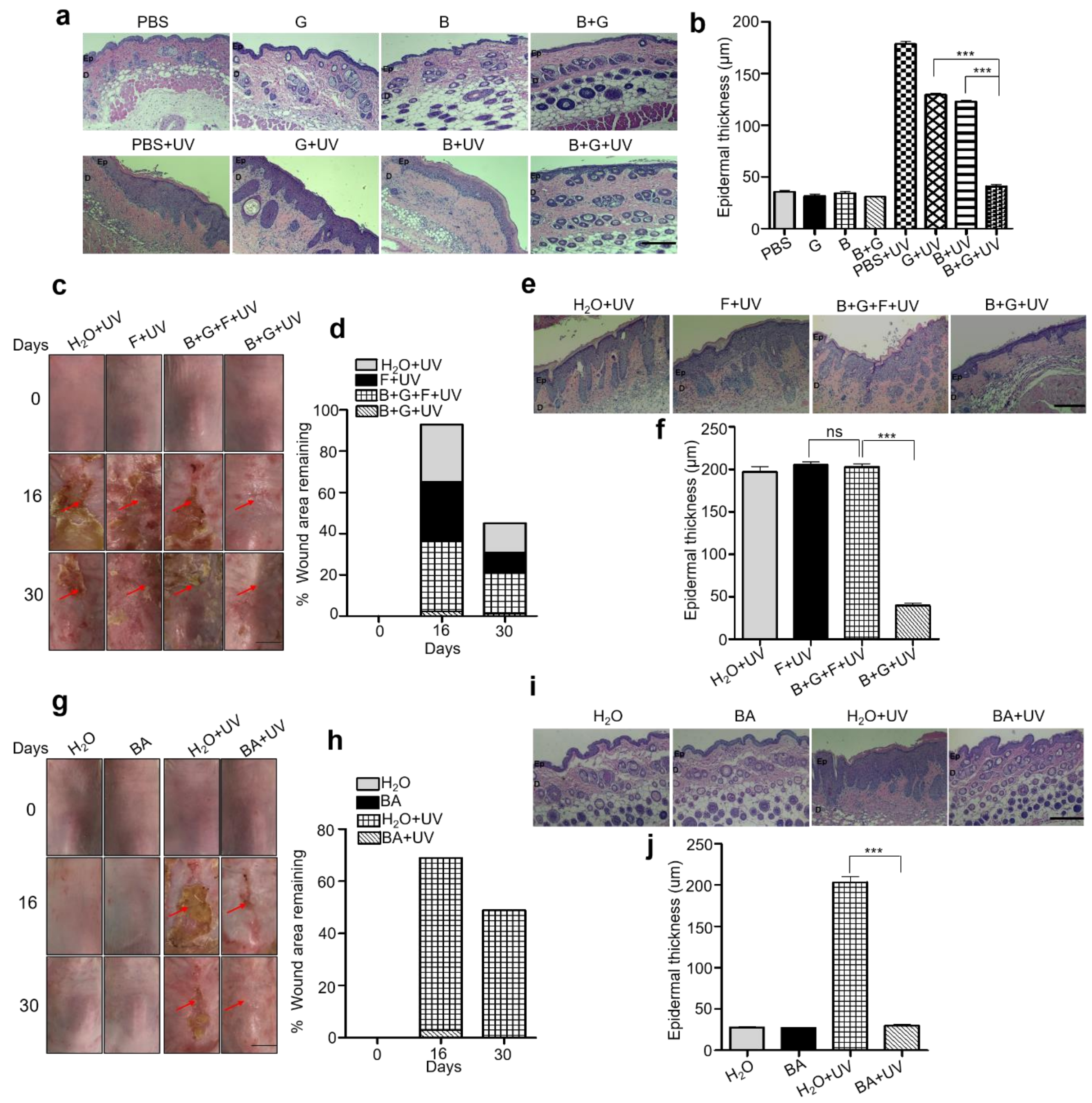

Figure S3. Attenuation of UVB-induced inflammation by application of a mixture of *S. epidermidis* and glycerol and its fermentation metabolite, butyric acid by decreased epidermal hyperplasia and improved skin morphology. (a) H&E staining (×100), showing epidermal thickness in different groups of skin topically applied with PBS, glycerol (G) (2%) , *S. epidermidis* bacteria (B) (10^7 CFU/ml), or a mixture of *S. epidermidis* bacteria plus glycerol (B+G) followed by exposure with UVB (195 mJ/cm2) irradiation from 30 d. Graph (b) from quantification of epidermal thickness in μm in above groups of ICR mice skin were displayed. Bar = 100 μm. (c) Skin morphology of ICR mice topically applied with H_2O, Furfural (0.4%), mixture of *S. epidermidis* bacteria (B) (107 CFU/mL), glycerol (G), (2%) and furfural (F) (0.4%) (B+G+F), and a mixture of *S. epidermidis* bacteria plus glycerol (B+G) followed by exposure with UVB (195 mJ/cm^2) irradiation were shown from 0, 16, and 30 d. Skin lesions or wound are indicated by red arrows. Scale bar = 5 mm. Graph (d) indicates percent of remaining wound area to the total area of exposed skin from all groups.(e) H&E staining (×100), showing epidermal thickness in skin from all groups. Graph (f) from quantification of epidermal thickness in μm from H&E staining in all groups of skin were displayed. Bar = 100 μm. (g) Skin morphology of ICR mice topically applied with H_2O and butyric acid (BA) (4 mM) followed by exposure with UVB (195 mJ/cm^2) were shown from 0, 16, and 30 d. Skin lesions or wounds are indicated by red arrows. Scale

bar = 5 mm. Graph (h) indicates percent of remaining wound area to the total area of exposed skin. (i) H&E staining (×100), showing epidermal thickness in skin from all groups. Graph (j) indicates quantification of epidermal thickness in μm from H&E staining in all groups of mice skin. Scale bar = 100 μm. Data are the means of three separate experiments using five mice per group. ns = non-significant. *** $p < 0.001$. (two-tailed t-tests).

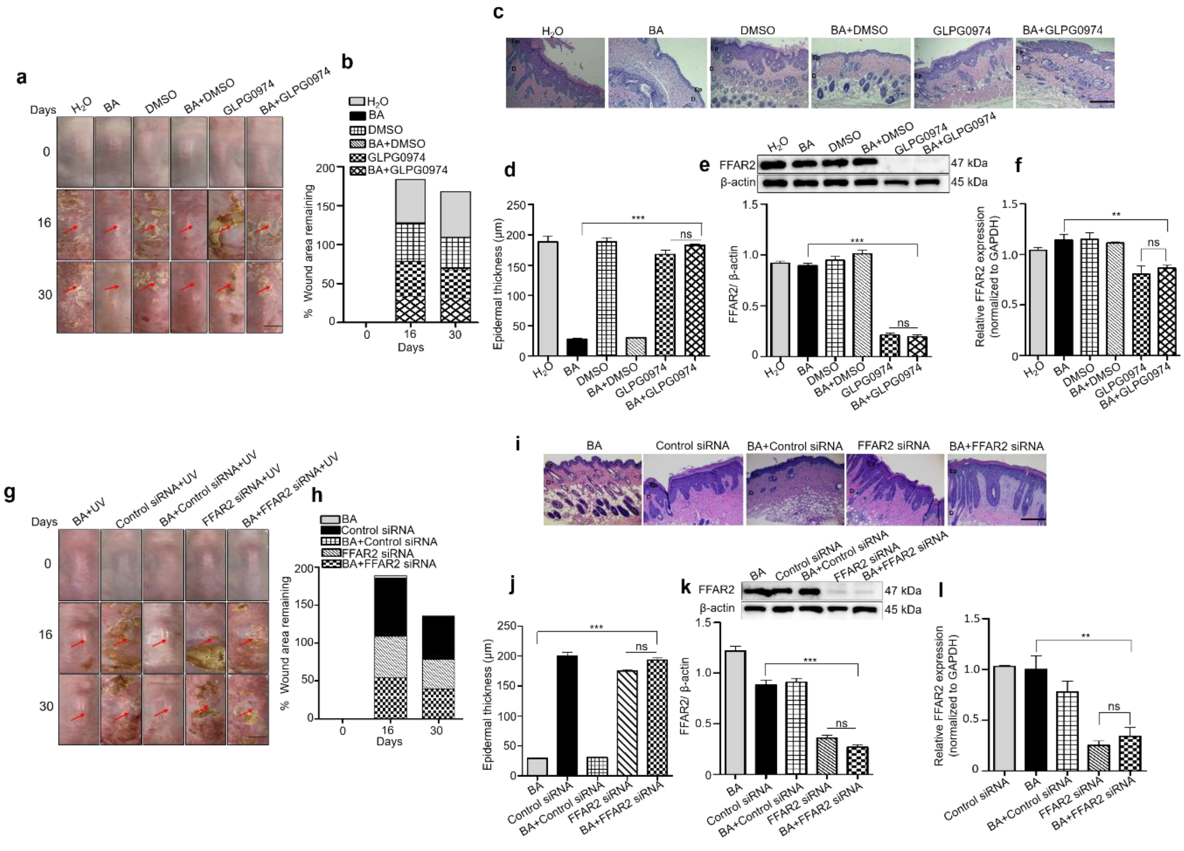

Figure S4. Blocking FFAR2 prevents the butyric acid mediated amelioration of skin lesions and epidermal thickness. (a) Skin morphology of ICR mice upon gavage feeding of FFAR2 antagonist GLPG0974 dissolved in DMSO in saline (0.01%) and topically applied butyric acid (BA) (4mM) followed by UVB irradiation (195 mJ/cm2) were displayed from 0, 16 and 30 d. Mice fed with DMSO in saline and topically applied with H_2O, followed by UVB exposure were included as control. Skin lesions or wound are indicated by red arrows. Scale bar = 5 mm. Graph (b) indicates percent of remaining wound area to the total area of exposed skin from all groups. (c) H&E staining (×100), showing epidermal thickness in skin from all groups. Graph (d) from quantification of epidermal thickness in μm from H & E staining in all groups of mice skin were displayed. Scale bar = 100 μm. (e) Protein expressions of FFAR2 and β-actin by western blot analysis in mice skin from all groups. (f) The expression of the FFAR2 gene relative to the GAPDH gene by RTPCR analysis in mice skin from all groups. (g) Morphology of ICR mice upon subcutaneous injection with FFAR2 siRNA 10 min prior to topical application with butyric acid (BA) (4mM). Injection with scrambled or negative control siRNA and topically applied with H_2O were included as control. Skin lesions or wound were indicated by red arrows. Scale bar = 5 mm. Graph (h) indicates percent of remaining wound area to the total area of exposed skin of mice from all groups. (i) H&E staining (×100), showing epidermal thickness in skin from all groups. Graph (j) from quantification of epidermal thickness in μm from H&E staining in all groups of mice skin are displayed. Scale bar = 100 μm. (k) protein expression of FFAR2 and β-actin by western blot analysis in skin from all groups. (l) The expression of the FFAR2 gene relative to the GAPDH gene by RTPCR analysis in mice skin from all groups. Data are the means of three separate experiments using five mice per group. ns= non-significant. * $p < 0.01$. (two-tailed t-tests).
